# Emergency remote learning during the pandemic from a South African perspective

**DOI:** 10.1007/s40979-021-00087-5

**Published:** 2021-11-02

**Authors:** Rashri Baboolal-Frank

**Affiliations:** Department of Procedural Law, Faculty of Law, University of Pretoria, Private Bag X20 Hatfield, Pretoria, South Africa

**Keywords:** Emergency remote learning, Experiential learning, Pandemic, South Africa

## Abstract

The COVID-19 pandemic created a situation for the implementation of emergency remote learning. This meant that as a lecturer at a traditionalist University of contact sessions, the pandemic forced us to teach remotely through online methods of communication, using online lectures, narrated powerpoints, voice clips, podcasts, interviews and interactive videos. The assessments were conducted online from assignments to multiple choice questions, which forced the lecturers to think differently about the way the assessments were presented, in order to avoid easy access to answers found in a textbook and online. This meant that more application questions of theory to practice were assessed in a more challenging way to prevent cheating and collaboration with peers. Formal assessments completed during emergency remote learning, have become the past practice, as innovative methods have been adopted for learning and for assessment purposes in order to preserve the integrity and attainment of the degree through online modes of learning. The aim of the paper investigates and explores the methods of teaching, together with the results obtained from the students of 2019 and 2020 in their final year relating to two final year modules against the literature relating to learning processes and methodologies.

## Introduction

The aim of this paper elucidates the traditional modes of assessments versus the emergency remote learning methodologies during the pandemic. The paper discusses the applicable literature against the results and postulates that students’ results did not improve during emergency remote learning assessments as there were other factors that affected their learning processes. The University of Pretoria in South Africa is a contact traditional tertiary institution. The study illustrates those students fared better in the traditional form rather than under the emergency remote learning methodologies relating to the online assessments. There are various reasons for that, in that over the years’ students have become accustomed to the traditional form of teaching, as well as consulting in physical form. The google meet and virtual classrooms, does not replace the gap that holistic physical contact possesses in relation to real time thinking. It is evident that learning at one’s leisure, which often leads to complacency. The competitive edge against peers is lost in the space of internet connectivity issues because fibre and data are expensive, home based learning environmental challenges, more responsibilities of helping out younger siblings with their schooling on the online flipped classroom. Since the COVID-19 virus has mutated into various strains, we are still learning about the symptoms and spread of the virus, online teaching is fast becoming the new normality for both the student and lecturer’s protection against the virus.

### Assessment results

The assessment results are compared against the traditional mode of learning versus the online learning modality results. Two final year subject results are considered in the 4th year LLB curriculum, namely an elective module of alternative dispute resolution and the final year compulsory module of civil procedure. The tabulated figures illustrate the total number of students for the year as well as the assessment in relation to the exams. The assessment for the module civil procedure in 2019 constituted of two semester tests, an online quiz and an exam. The assessment for 2020 followed the same assessment criteria however, the exam was conditional, meaning, that if an assessment overall average of 65% was obtained, the student was exempt from writing the exam, which resulted in a 97% promotion of the number of students, being exempt from writing the exam. Of the 13 students that wrote the exam, 8 passed and the resulting four did not and qualified for a supplementary exam, where 3 passed with one failure. Whereas in 2019, all the students passed the exam and there were no supplementary exams.

### Tabulated results

**Table:** 

Year	2019	2020
Civil Procedure	474 students	471 students
Exams	474 students	13 students
Supplementary exams	none	4 students
Failures	none	1 student
Alternative Dispute Resolution	86 students	126 students
Exams	86 students	2 students
Supplementary	none	none

The table for alternative dispute resolution, illustrates that in 2019 there was a total of 86 students, and that all students passed the exam successfully, taking away the need for a supplementary exam. In 2020, there were a total of 126 students, with a 98% promotion rate of exemption from the exam. The two students that wrote the exam passed successfully, and as a result, there were no supplementary exams written. There were thus no failures in both years. The assessment opportunity for 2019 consisted of an oral component, semester test and online quiz. The assessment opportunity in 2020 was the same, except all forms of assessment were online. This online assessments includes the semester test being multiple choice, consisting of matching columns. This form of assessment is unlike the traditional framing of questions which is usually essay questions and short answers prior to the implementation of online assessments. The rationale for avoiding short answer and essay type questions is to prevent copying and to ensure that students think quickly relating to application questions, and protecting the values, integrity and ethics of independent studying as a critical thinker.

## Literature review

There has been a trend and need for online teaching methodologies in the teaching of law. In order to modernise the curriculum in accordance with the fourth industrial revolution, it is necessary for the law schools to move away from traditional contact sessions. (McGrath et∼al. 2020 p.6) McGrath et∼al. (2020 p.6) argues that it is evident that more law schools are utilising web-based multimedia material to improve the quality of legal education. According to the literature study conducted by McGrath et∼al. (2020 p.6) there have been an array of mediums for improving the classroom experience and replace the flipped classroom experience with computer-aided tools for law students as well as digital learning resources to ensure a safe training environment for the students. The digital learning resources provides a platform to utilise and mobilise the knowledge acquired by students. (McGrath et∼al. 2020 p.6) Professor Crick (2021, p.16) expounded that the COVID-19 pandemic was the catalyst for change in digital education. These propositions align with the view that Covid-19 provides an opportunity for more opportunities for creation into a new realm of digital education, with your work environment being at home. The literature also explores other theories that supports the theory of learning in the digital age of the pandemic.

Rowland and Hall postulate the exploration of organizational learning through recognised management systems that are responsible for contributing to organizational effectiveness and competitive advantage. (2014 p.342) There have been numerous authors that have contributed to the intellectual caucus of organizational learning prior to the 1990s such as Argyris and Schon (1978), Cyert and March (1963), Simon (1955), Kolb and Fry (1975), Lewin (1951), Emery and Trist (1960) and the Tavistock Institute (Miller 1976; Rice 1965) (Rowland & Hall 2014,p. 343) The approach relating to systems thinking that Schein (1965 p.95) adopts consists of “open systems concepts of constant interaction, multiple goal seeking, interdependence, the input-transformation-output model, subsystems and system boundaries in his redefinition of organization in terms of processes.” The adaptation to the environment circumstance is about maintaining a dynamic equilibrium through homeostasis. (Rowland & Hall 2014 p. 344) Another aspect that is essential to organizational learning is the different levels of learning. (Rowland & Hall 2014 p. 344) Chris Argyris (1977) described the organizational process as “a process of detecting error”. Rowland & Hall expounds that the process relates to any aspect that hinders learning. (2014 p. 344) When the process facilitates the organization to continue its goals, objectives and present policies, the process is termed “the single loop learning.” (Argyris 1977 p.116) Argyris developed the double loop learning and theories with Donald Schon (Argyris and Schon 1974). Single loop learning identifies errors and proposes the solutions. (Rowland & Hall 2014 p. 344) Double loop learning focuses on the values and assumption and is the process to challenge the fundamental norm and aims together with exposing the differences between theories in use and adopted theories. (Rowland & Hall, 2014 p. 344) The double learning loop has been described as “holistic, adaptive and future oriented.” (Rowland & Hall 2014 p. 344) Broersma (1995) has described the double learning loop as systemic learning. The triple loop learning was discussed by Tosey et al. (2012). The triple loop learning is described “as deuteron learning” by Bateson (1973) and termed as “transformational learning” by Broersma (1995). (Rowland & Hall 2014 p. 344) The triple learning loop focuses on the process of how to learn, reflection on the previous constructs and conditioning of learning and devising and creating new learning strategies to overcome the barriers to learning. (Rowland & Hall 2014 p. 344) Rowland & Hall (2014 p.344) postulates that the process aims to develop a tolerance for failure and to acknowledge that the outcome and the expectations are not synchronized, hence fostering the skill of resilience. Rowland & Hall (2014 p.344) further espouses to challenge the theories relating to collaborative review and encouraging inquiry and reflection relating to the learning process. The psychological definition of learning is that it is “any relatively permanent change of behaviour that occurs as a result of experience.” (Collins 2012) Individual learning utilizes cognitive, effective and behavourial domains. (Rowland & Hall 2014 p. 344) The measurement of organizational learning is against performance and reward. (Rowland & Hall 2014 p.346–347) Similarly with organizational learning, tertiary education also creates a similar stimulus for learning in and beyond the classroom in relation to engaging with peers, the study material and experts in the field relating to complex subject matter for deconstruction and analysis. The theory of organisation learning is important as it illustrates the different learning complexities that students explore in an educational context, that prepares them for an organisation context of a law firm.

Strategic quality and management and human resource alignment have aimed to improve the performance of the organization through the development of people as human capital. (Rowland & Hall 2014 p.346) Similarly, the value that is placed on the student’s quality of production in their results and work that is produced, provides them with employment opportunities. When we engage with the employees of the organisation, we gauge the challenges and assist them to effectively overcome challenges and contribute to the bottom line of the organization. (Rowland & Hall 2014 p. 346) Positive engagement with students, reaps benefits for both the University and lecturer, to understand the needs of the students in their lack of performance. Reward is an expansive concept in that it stretches the dimensions of personal growth and autonomy. (Rowland & Hall 2014 p.347) There are other theorists that state that models of motivation, like Maslow’s self-actualisation, assist with the learning process and personal growth and development. (Maslow 1943) The research conducted by Seligman (2011) postulates the idea of positivity and introduces the acronym of PERMA, which stands for Positive emotion, Engagement, Relationships, Meaning and Achievement. (Rowland & Hall 2014 p.347) He hypothesises that happiness is what people seek in the workplace. (Rowland & Hall 2014 p.347) Fisher (2010) similarly argues for happiness at work, which determines the loyalty and commitment of employees to the organization. (Rowland & Hall 2014 p.347) The development of organization management and learning theories is continuously evolving for the enhancement of performance. (Rowland & Hall 2014 p.354.) Happier employees are more productive in the workplace, similarly the development of the app, and working for reward would motivate employees to ensure it is a success. Likewise, students gain their reward through the marks that they receive, in order to gain experience into a good firm after attaining their degree, as the market is incredibly competitive when it comes to securing articles for prospective candidate attorneys. A positive engaging environment, creates a safe space for the students to freely engage with their peers and lecturers, creates an ideal opportunity for the learning and development of the students. When a student is happy and motivated, they are able to overcome any challenge that is in their path of learning.

**Fig. 1:**
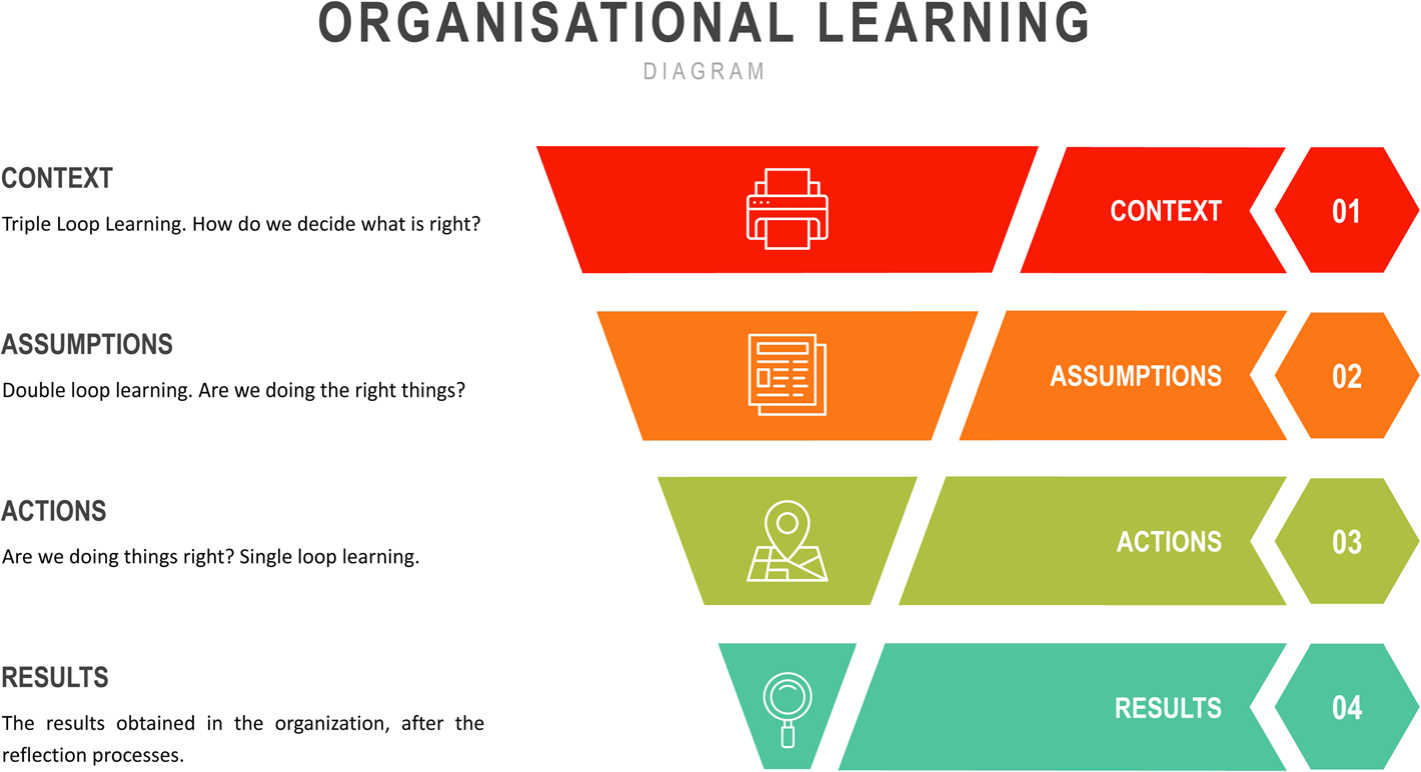
Adaptation of Thorsten.org (Rowland & Hall 2014 p.345)

Figure 1 illustrates the importance of the different learning loops, that is applicable to student learning. The triple loop learning consists of determining ‘what’ is right, in problem solving, that is the correct approach to determine the right answer. The double loop learning provides for inquiring the assumption whether the student is actioning the right thing in relation to problem solving and cognitive thinking. The single loop thinking, before even acting, the student ponders whether their approach to the question is correct, that is the application of the theory to the problem posed.

An investigation was conducted into research ethics and integrity through a case study of interviews with researchers and it was concluded that research ethics and integrity are formulated through experience, conditioning, development, mentorship and values that have been inculcated in childhood phase and through sport and by parental guidance. (Satalkar & Shaw 2019) It is apparent that effective communication is pivotal to the successful completion of the project. (Mountain and Davidson, 2011) The complexities of the organization, require time, patience, consultation and an innovative approach to study it thoroughly to understand the intricacies, mechanisms and movement of the organization to obtain success. (Pavlovska 2013) Kahler’s five drivers of human behaviour are observable in distancing language namely, “Be strong”, “Try hard”, “Be perfect”, “Hurry up” and “Please me”. (Kahler 1975) Tudor (2008) postulates the sixth driver as “Take It”. The driver messages are to encourage reaching one’s goals despite the circumstances. (Tudor 2008 p.53) These aspects encourage the management of employees of the company to adopt a positivist approach in the adoption of a mobile app. Hugman proposes that the modern ethical practitioners display various characteristics relating to discursive code of ethics. (Hugman 2003 p.1037) The aspects that are displayed is on principles and values. (Hugman 2003 p.1037) The embracing of diversity in all forms is a characteristic that is imbibed by a morally active practitioner. (Hugman 2003 p.1037) The internal struggles between the individualist goals versus the collective goals in an organization. (Hugman 2003 p.1037) To support others to be morally active and encourage a strong code of values and rules. (Hugman 2003 p.1037) The recognition of the contextual nature of the practice in relation to maintenance, change and caring of others and the organisation. (Hugman 2003 p.1037) Social discrimination of minority groups are the unfair treatment based on prejudice. (Sarantakis 2017 p.135) Cognitive discrimination is when psychological and pragmatic determinations is placed into specific categories to demonstrate biased opinions and judgments of specific groups. (Sarantakis 201, p.135) The behaviour of cognitive and social discrimination is the manner of reasoning that one makes daily decisions and thus is intertwined with behaviours and patterns of individuals. (Sarantakis 2017 p.135) The behaviour and practices of anti-discriminatory practices is dictated by awareness and critical thinking of self-reflection of one’s behaviour and choices, together with the knowledge of legislation that prevents discriminatory practices and behaviour in organisations. (Sarantakis 2017 p.139) There should be active voices of the management and leadership in support of anti-discriminatory practices and behaviour, which sets the example for all employees to follow. (Sarantakis 2017 p.139) Similarly, effective communication is pivotal for learning, all voices of the students must be heard by the lecturers, regarding the different needs and wants in order to engage with the subject material. It is necessary for all students to engage without fear or prejudice or the fear of being discriminated against, in the creation of a safe space of learning online or virtual is quintessential.

Driscoll and Allan (2014) suggest that the implementation of reflective writing has the ability to enhance learning outcomes, fostering student learning and engaging the faculty in professional development. The research-based assessment process assists the students to gain profound insights. (Allan & Driscoll 2014) Research based learning further promotes metacognition and transfer of learning that allows for professional development. (Allan & Driscoll 2014) “When students reflect upon their learning, they engage in a potentially transformative act of responding to, connecting with, and analyzing an experience, event, process, or product.” (Allan & Driscoll 2014 p.37) Reflection is a process that allows for thought and action to synchronise. (Allan & Driscoll 2014 p.37) An opportunity is provided to students to reflect upon their internal processes, criticize their challenges and celebrate their successes and achievements. (Allan & Driscoll 2014 p.37) This process is transcribed into words that allows them an opportunity to self-reflect onto paper. (Allan & Driscoll 2014 p.37) Dewey postulated that reflective thinking culminates into powerful educational transformations. Reflective practices provide an opportunity for the student to engage with learning in a very fluid manner. (Allan & Driscoll 2014 p.38) The primary purpose for learning is to allow students to imbibe the learning lessons into a variety of contexts such as personal, professional, educational and civic contexts. (Perkins, Tishman, Ritchart, Donis, & Andrade 2000; Russel & Yanez 2003) Reflection is empowering for the student and it allows them to deconstruct the learning process and plan their future learning contexts. (Allan & Driscoll 2014 p.39) Reflection allows the student to engage and examine the work that they have learnt in a personal manner that allows for critical self-review of the discipline learnt together with the learning outcomes. (Allan & Driscoll 2014 p.50) The student also has another method to take accountability of their studies by allowing for metacognition relating to the challenges they confronted and the solutions for overcoming these challenges successfully. (Allan & Driscoll 2014 p.50) This exercise of reflective writing is becoming more important in the virtual space to obtain the feedback from students relating to the subject matter from a critical discourse.

We live in a society that is evolving faster than we can keep up as humans are constantly forced to evolve with the times. Transformative learning is a method of adapting the learning processes to correlate with the environmental changes of teaching. (Illeris 2014 p.573) The individual personality keeps evolving with the present context of change, one has to constantly digest the emotions and environment and elect a path forward as a coping mechanism, which marries into transformative learning as the personality transformation is interlinked to one’s learning context. (Allan & Driscoll 2014 p.579) Transformative thinking for law students and lawyers must become the norm to adapt to versatile circumstances.

**Fig. 2:**
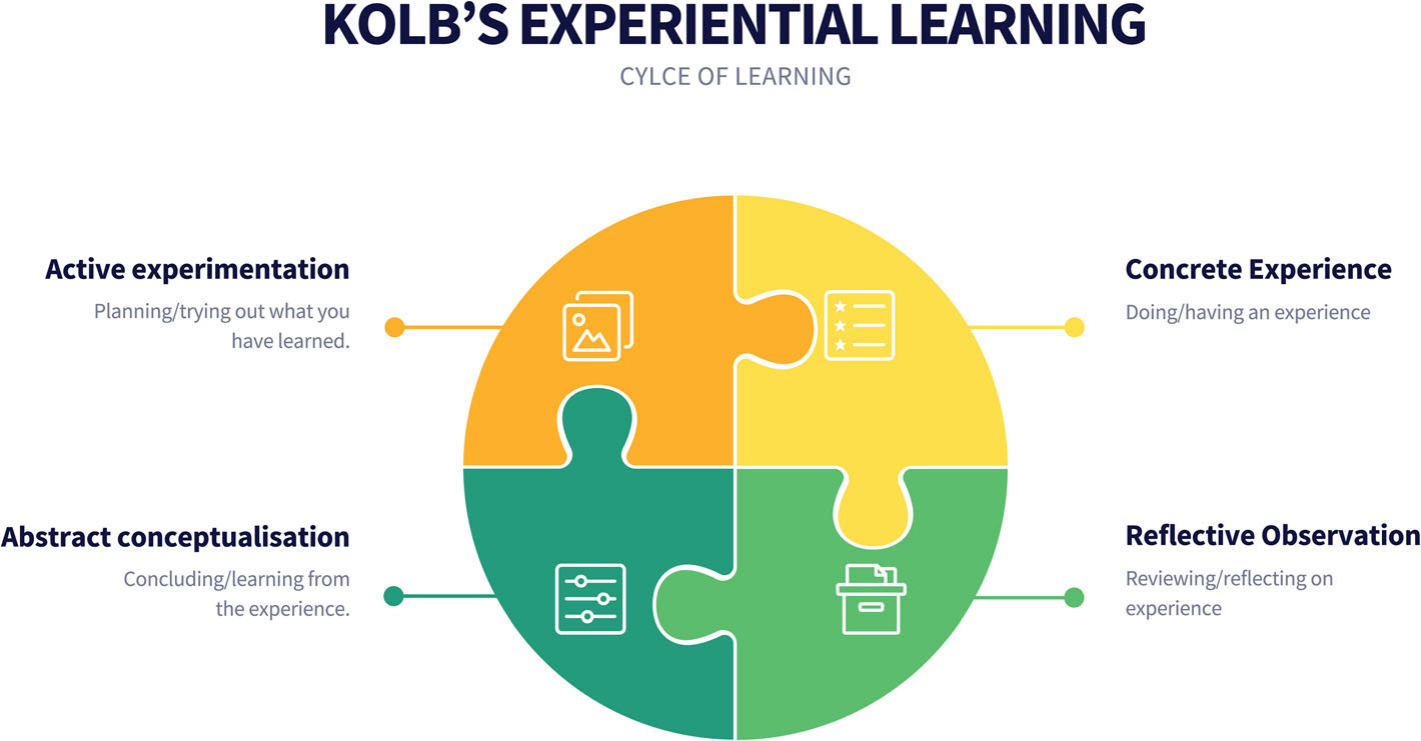
Adaptation: Kolb’s experiential learning (1984)

Kolb’s experiential learning for reflective learning is distinctive from an accommodative learning style (see Fig. 2). (Li & Armstrong 2015 p.422) Kolb’s (1984) experiential learning theory (ELT) is defined as:

“...the process whereby knowledge is created through the transformation of experience. Knowledge results from the combination of grasping and transforming experience.” (p.41)

Kolb’s ELT relates to two dimensions namely the abstract concrete dimension and the theoretical concepts that grapple with tangible new experiences. (Li & Armstrong 2015 p.423) It is apparent that the personality is different from the ELT, as these aspects are different, however the ELT affects one’s personality. When applying Kolb’s experiential learning cycle to practice it is apparent that concrete experience, provides for students taking action in carrying out tasks and exercises of the courses. The reflective observation allows students to reflect upon the actions of the learnings processes and assessments of the course. The abstract conceptualization allows us to learn from the experience, relating to the challenges that students confronted in the courses. The active experimentation allows students to apply all the theoretical knowledge that they have gained through traditional forms of study and apply it to practice by doing the respective tasks of the courses to achieve the objectives and outcomes of the courses.

## Conclusion

It is apparent that the theory relating to organisational learning is applicable in relation to traditional learning. Kolb’s experiential learning cycle is still relevant for tertiary students in 2020. The navigation from traditional modes of learning to online learning is not an easy leap, yet it is still very possible, and students still excel overall from the results that were produced. However, the protection of integrity, values, respect, independent learning becomes a challenge that is constantly evolving in relation to more challenges that appear in different forms as barriers to learning and teaching. It is quintessential for both the lecturer and the students to be equipped to mentally and physically adjust to the new normality of online tertiary education, with a renewed vigour, passion, grit and pursuit for the learning experience of gaining an education.

## Data Availability

Not applicable. I do not wish to share the data because it is confidential information of the students.

## Abbreviations

ELT: Experiential Learning Theory
LLB: Legum Baccalaureus/Bachelor of Laws
PERMA: Positive emotion, Engagement, Relationships, Meaning and Achievement
